# Can Individual Movement Characteristics Across Different Throwing Disciplines Be Identified in High-Performance Decathletes?

**DOI:** 10.3389/fpsyg.2020.02262

**Published:** 2020-09-18

**Authors:** Fabian Horst, Daniel Janssen, Hendrik Beckmann, Wolfgang I. Schöllhorn

**Affiliations:** ^1^Department of Training and Movement Science, Institute of Sport Science, Johannes Gutenberg-University Mainz, Mainz, Germany; ^2^Gymnasium Dionysianum, Rheine, Germany

**Keywords:** motor learning, pattern recognition, high-performance sports, machine learning, support vector machine, individuality, transdisciplinary individuality

## Abstract

Although the individuality of whole-body movements has been suspected for years, the scientific proof and systematic investigation that individuals possess unique movement patterns did not manifest until the introduction of the criteria of uniqueness and persistence from the field of forensic science. Applying the criteria of uniqueness and persistence to the individuality of motor learning processes requires complex strategies due to the problem of persistence in the learning processes. One approach is to examine the learning process of different movements. For this purpose, it is necessary to differentiate between two components of movement patterns: the individual-specific component and the discipline-specific component. To this end, a kinematic analysis of the shot put, discus, and javelin throwing movements of seven high-performance decathletes during a qualification competition was conducted. In total, joint angle waveforms of 57 throws formed the basis for the recognition task of individual- and discipline-specific throwing patterns using a support vector machine. The results reveal that the kinematic throwing patterns of the three disciplines could be distinguished across athletes with a prediction accuracy of up to 100% (57 of 57 throws). In addition, athlete-specific throwing characteristics could also be identified across the three disciplines. Prediction accuracies of up to 52.6% indicated that up to 10 out of 19 throws of a discipline could be assigned to the correct athletes, based on only knowing these athletes from the kinematic throwing patterns in the other two disciplines. The results further suggest that individual throwing characteristics across disciplines are more pronounced in shot put and discus throwing than in javelin throwing. Applications for training and learning practice in sports and therapy are discussed. In summary, the chosen approach offers a broad field of application related to the search of individualized optimal movement solutions in sports.

## Introduction

Most of us are familiar with the experience of identifying friends or colleagues by their walk (Cutting and Kozlowski, 1977), even from a distance and with limited visibility (Stevenage et al., 1999). Practitioners in the field of sports science and physical therapy often report the same experience of identifying individual athletes or patients based on their movement characteristics (e.g., a characteristic forehand stroke in tennis or a unique hand movement). Additionally, most of us have observed people mastering certain tasks easily, while struggling to become proficient in others. Both experiences serve as evidence of the individuality of human movements, though they may hold various meanings and act epistemologically on different time scales (Newell et al., 2001). The tacit, universal acceptance of movement as a method of identifying individuals suggests that most of us understand individualized movement, yet the perfunctory nature of this acceptance has inhibited a deeper investigation of the concept’s essential aspects and consequences.

In human movement science, anecdotal evidence has made claims of “individuality” for years (Bernstein, 1967; Marteniuk, 1974). Although the importance of individuality in sports training has been recognized since the origins of sports science (Matveev, 1970), the phenomenon has been mostly regarded as a negligible side effect or as an exception in the search for universal scientific laws (Harre, 1969, 2013; Matveev, 1970; Huber, 1977; Schmidt and Young, 1991; Nitsch et al., 1997; Schnabel et al., 1997). In most cases, individuality appeared in the context of reliability studies that compared intra- and inter-individual variance (Bates et al., 1983). These reliability studies led to the standard requirement that an average of 10 to 25 movement trials be conducted for each individual participant to achieve an appropriate level of reliability or reproducibility (Bates et al., 1983; DeVita and Bates, 1988; Gollhofer et al., 1990). The extent to which the inter-individual variance distributions overlap to discriminate individuals from one another was not investigated.

In the past, the term individuality most often has been normatively applied in three ways: (1) when no classification criteria could be found (Brüggemann et al., 1991; Schöner et al., 1992; Button et al., 2000; Hecksteden et al., 2015; Barth et al., 2019), (2) to explicitly circumvent “the difficulty of achieving statistical significance” by creating smaller standard deviations and by describing several single cases (Davids et al., 1999; Button et al., 2000; Nuzzo, 2014), and (3) when individuality was predetermined in the form of case studies (Mendoza and Schöllhorn, 1990; Schöllhorn, 1993, Schöllhorn, 1998; Wallace et al., 1994; Bauer and Schöllhorn, 1997; Button et al., 2006; Chow et al., 2006). Frequently, all three interpretations were used in combination and reflected a rougher approximation of the phenomenon than scientific evidence would suggest.

While movement and sports science still struggle to balance the demands of group-oriented science and individual athlete- and patient-oriented practice, forensic science remains primarily concerned with individual cases that must lend legal validity. Therefore, the field of forensic science has developed specific methods and criteria for the identification of individuals (disjunct separation) (Kaye, 2010). In this context, Jain et al. (2006) suggested that before individuality can be assumed, one must test the probability of uniqueness (indicating that no two persons have identical characteristics) and the persistence/permanence of a physiological or behavioral characteristic (meaning that the characteristic should be invariant with time).

The first steps toward such criteria took place in the analysis of everyday and sports movements and revealed the identification of individual people based on gait (Schöllhorn et al., 1999, 2002; Nixon et al., 2006), running (Simon and Schöllhorn, 1995), pole vaulting (Jaitner and Schöllhorn, 1995), discus (Bauer and Schöllhorn, 1997), and javelin throwing (Schöllhorn and Bauer, 1998). The proposed approaches used self-organizing Kohonen maps, in combination with cluster analysis, as early representatives for machine learning classification in human movement science. The results indicated the structural application of a statistical approach that is, similar to forensic proceedings, oriented toward a generic understanding of probability. As follows, the probability of an event occurring is equal to the number of ways of achieving success relative to the possible number of outcomes. For example, Schöllhorn and Bauer (1998) recorded 10 javelin throws by a single athlete at different competitions over 5 years. Subsequently, the kinematic patterns of these 10 throws were clustered together out of 51 kinematic patterns of throws from several other athletes. The probability of achieving this classification by chance is extremely low (<1 × 10^–17^). This outcome is far below the magnitude of common probabilities used, first, in the statistical model based on the work of Fisher and Mackenzie (1923) or Neyman and Pearson (1928) and, second, in the magnitude of becoming legally relevant.

Meanwhile, the uniqueness and persistence of individual movement patterns could be validated for versatile whole-body movements such as walking (Horst et al., 2017b, 2019), pedaling (Hug et al., 2019), basketball throwing (Schmidt, 2012), horse riding (Schöllhorn et al., 2006), or playing a musical instrument (Albrecht et al., 2014). Up to this point, one might still be tempted to argue that a single movement pattern is optimal for an individual athlete. Initial doubts were raised, however, when it was remembered that none of the aforementioned studies could demonstrate identical repetitions of movement patterns, and thus strong indications were provided for the intuitively assumed (Bernstein, 1967) and previously biomechanically derived (Hatze, 1986) extremely low probability of identical repetitions. Theoretically, however, the continuous fluctuations that were observed during the proof of persistence could have been due to limitations in the biomechanical measurement resolutions or could have simply been random, unstructured noise. More detailed investigations of fluctuations between repeated movement executions within individual persons surprisingly revealed strong evidence of fine structures within a class of movement patterns. These finer structures showed a dependence on fluctuations in emotion (Janssen et al., 2008; Janssen, 2017), on fatigue (Jäger et al., 2003; Janssen et al., 2011), or on time (Bauer and Schöllhorn, 1997; Schöllhorn et al., 2002; Rein et al., 2010) with different time scales (Horst et al., 2016, 2017a). The individuality of movement patterns in connection with their fine structures thus indicate that individual movement patterns continuously change and adapt over time.

In practice, the identification of athletes based on their individual movement patterns and their corresponding fine structure does not require individually tailored learning or training methods. If we assume that individual differences exist from birth, then theoretically, the same learning and training content could have led to individually distinguishable movement patterns at a later age. However, this awareness is subject to the assumption that everyone responds in the same way to intervention measures. To shed more light on these questions, further studies on individual learning behavior should be conducted.

Indications for individual responses on a similar intervention came from physiological (Bouchard and Rankinen, 2001) and biomechanical studies (Cole et al., 1995; Schöllhorn et al., 2001, 2002). In the meantime, an increasing number of studies have observed phenomena that indicate the individuality of adaptations and learning (Schöllhorn et al., 2006; Kostrubiec et al., 2012; Caballero et al., 2017). References to the advantages of learning with individual role models also began to question learning approaches based on average-oriented group role models (Brisson and Alain, 1996). Despite these initial signs, scientific evidence of individual learning processes according to the criteria of uniqueness and persistence is still missing (Fisher et al., 2018). Because of the normative nature of these studies, a criteria-driven analysis, as proposed by Jain et al. (2006), is suggested. Applying the same criteria of uniqueness and persistence to motor learning and adaptation processes requires, first, that each athlete/patient (e.g., in terms of changes in movement patterns or performance outcomes) respond differently to a particular intervention (uniqueness) and, second, that individual responses to multiple interventions can be repeatedly demonstrated (persistence).

While the first criterion could be tested indirectly via the degree of learning progress each participant achieves, testing the criterion of persistence is more complicated. The simplest way to prove the persistence of individual learning characteristics would be to allow athletes to learn the same skill several times after wash-out phases. However, a limitation with this approach has been raised by re-learning studies (Newell et al., 2001; Liu et al., 2003). Once a movement is acquired and forgotten, it is re-learned more quickly (Malone et al., 2011). In consequence, initial learning conditions cannot be reproduced exactly when a skill is re-learned several times, even with adequate wash-out phases. This fact makes it almost impossible to compare the persistence of learning processes adequately. Alternatively, individual characteristics of learning processes should be observable in the acquisition of different skills. Therefore, finding approaches that can detach the individual-specific characteristics from the task-specific characteristics in various movements would be helpful. Interestingly, previous studies on the uniqueness and persistence of movement patterns have only been carried out within a single movement task.

This pilot study aims to analyze the three throwing disciplines in the decathlon (shot put, discus throw, and javelin throw) and to search for athlete- and discipline-specific similarities in the kinematic throwing patterns of these disciplines using automatic classification by means of machine learning. Instead of merely testing the individuality of athletes in a single throwing discipline, the present classifications are used to test whether the kinematic throwing patterns will be assigned to the correct throwing discipline and whether the knowledge of, for example, an individual athlete’s shot put movement patterns predicts the individual’s discus or javelin movement patterns. For this objective, high-performance athletes competing in a national decathlon qualification competition were selected. This high performance level served to guarantee sufficient stability for all three throwing movements. A competition was selected for this study because it is a setting in which athletes often demonstrate their best performances, which we assume can increase the expression of the individuality in their movement patterns (Schöllhorn et al., 2002).

## Materials and Methods

Seven right-handed, male decathletes (18.9 ± 0.4 years), who were members of the German junior national team with at least 5 years of experience in the decathlon, were recorded during a national decathlon qualifying competition. The final throwing phases of 19 shot puts, 19 discus, and 19 javelin throws were analyzed (Table 1). For right-handed athletes, the final throwing phases all begin when the left foot touches down and end when the throwing object is released from the hand. Most of the increase in velocity of throwing object is produced during this phase (Hay, 1993; Bauersfeld and Schröter, 1998). The throwing performances ranged from 11.70 to 15.06 m (shot put), 33.66 to 43.74 m (discus), and 40.08 to 58.03 m (javelin).

**TABLE 1:** Number of throwing trials per athlete and discipline.

Athlete	Age (years)	Shot put	Discus	Javelin
A1	18–21	3	3	3
A2	18–21	3	3	2
A3	18–21	1	3	3
A4	18–21	3	1	3
A5	18–21	3	3	2
A6	18–21	3	3	3
A7	18–21	3	3	3
Sum (disciplines)		19	19	19

The recordings were taken using two high-frequency video cameras (Weinberger MiniVis Eco-2; frequency: 200 fps; resolution: 1280 × 1024 pixel), which were positioned orthogonal to each other, one facing toward the flight direction of the throwing object. A space of 3 × 3 × 3 m^3^ was covered for the analysis using a calibration cube with 25 marker points. Due to the official competition rules, no marker points were allowed on the athletes. Data of both cameras were synchronized using an electric impulse transmitted from the master camera to the slave camera during each throw. Neither the athletes nor the experienced digitizers were informed about the aim of the study. The following anatomical body landmarks were digitized: the manubrium sterni, the left and right acromion, the epicondylus lateralis, the processus steyloidus ulnae, the spina illiaca anterior superior, the trochanter major, the lateral end of the femur (knee), the patella, the articulatio tibo fibulare talare, the calcaneus, the phalanx distalis, and the hallux.

The digitization of these points allowed the estimation of three-dimensional joint angles of the right and left shoulder, elbow, hand, hip, knee, and ankle. All videos were manually digitized with SIMI Motion Software 5.0 (SIMI Reality Motion Systems, Germany). Data were filtered using a recursive, second-order Butterworth filter with a cutoff frequency of 14 Hz. All trials were digitized for an additional 10 video frames at both phase boundary ends because of filter effects at the beginning and end of each signal.

For javelin, the duration of the final throwing phase lasts about 130 ms; for discus throwing, about 400 ms; and for shot put, about 200 ms (Ballreich and Kuhlow, 1986; Ballreich et al., 1989). Despite the variable duration of these final throwing phases, commonalities between all three throwing disciplines are assumed and used to economize training in combined events (Hay, 1993). The trials were time normalized to 26 intervals to compare the kinematic patterns of three disciplines. After time normalization, the amplitudes were normalized over all trials and all athletes into the interval [0;1].

**FIGURE 1:**
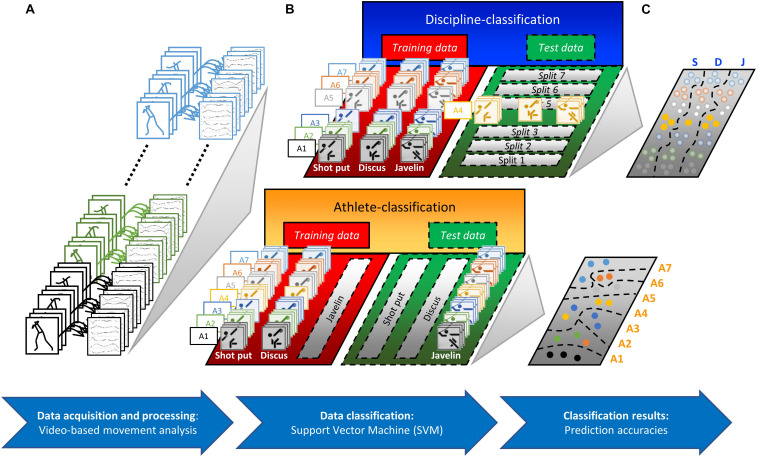
Schematic overview of our approach to data acquisition, processing, and classification. **(A)** Seven right-handed athletes (A1–A7), who were members of the German junior national team with at least 5 years of experience in the decathlon, were recorded during a national decathlon qualifying competition. The kinematic analysis included all valid trials of the competition in the three throwing disciplines: S, shot put; D, discus throw; J, javelin throw. The final throwing phases of 19 shot puts, 19 discus, and 19 javelin throws were analyzed using two orthogonally positioned high-frequency video cameras. After the digitization of 23 anatomical body landmarks, the three-dimensional joint angles of the right and left shoulder, elbow, hand, hip, knee, and ankle were estimated. **(B)** Time- and amplitude-normalized joint angle waveforms formed the input vectors for the classifications using a support vector machine (SVM). An athlete-classification using a leave-discipline-out cross-validation and a discipline-classification using a leave-athlete-out cross-validation were performed. **(C)** The performance of the classification models was assessed based on the prediction accuracy.

Time- and amplitude-normalized data formed the input vectors for the classifications using a support vector machine (SVM) (Cortes and Vapnik, 1995). The classification of the kinematic throwing patterns based on SVM represents a supervised learning approach for pattern recognition in data sets. The ability to distinguish kinematic throwing patterns was investigated in multi-class classifications using a “one-vs.-all” algorithm. The L2-regularized, L2-loss, support-vector classification of the Liblinear Toolbox 1.4.1 (Fan et al., 2008) was applied with a linear kernel function within the software environment Scilab 6.0.2 (Scilab Enterprises, France). A grid search within the range of C = 2^–5^, 2^–4.75^, …, 2^15^ was conducted to determine C experimentally before the training and testing of the SVM models. An athlete-classification using a leave-discipline-out cross-validation and a discipline-classification using a leave-athlete-out cross-validation were performed. This processing means that data from one discipline (in the case of the athlete-classification) or from one athlete (in the case of the discipline-classification) were used either as training or as test data during the cross-validation of the SVM models. A schematic overview of the entire approach with data acquisition, processing, and classification is depicted in Figure 1.

In the case of athlete-classification, the kinematic data of one discipline were used in the cross-validation, either as training or as test data (cf., athlete-classification in Figure 1). This use of data means that the classification model did not “see” the throwing patterns of the athletes in the tested discipline during the training process. In the first cross-validation split, the classification model was first trained to distinguish the athletes based on the normalized kinematic patterns of all shot puts and javelin throws. Then, the performance of the classification model was tested using the normalized kinematic patterns of all discus throws.

In the case of the discipline-classification, the SVMs were trained with the corresponding partitions of variable waveforms of all athletes (except one) in all three throwing disciplines. The remaining waveforms of the one athlete were used to test the performance of the SVM models for classification into one of the three possible throwing disciplines.

## Results

The results of the athlete-classification are shown in Table 2. When the classification models were tested with data from the discus throws, the results showed the highest prediction accuracy of 52.6% for athletes when the SVM considered all kinematic variables except the variables of the throwing arm. The lowest predictive accuracy of 21.1% was obtained when only the kinematic data of the lower-body joint angles were considered.

**TABLE 2:** Prediction accuracy of the athlete-classification with leave-discipline-out cross-validation for different data partitions.

Test data	Training Data	Random baseline	All variables	All variables (without throwing arm)	Only upper-body variables (without throwing arm)	Only lower-body variables
Discus	Shot put and Javelin	14.3% (1/7 athletes)	42.1% (8/19 test trials)	52.6% (10/19 test trials)	47.4% (9/19 test trials)	21.1% (4/19 test trials)
Shot put	Discus and Javelin	14.3% (1/7 athletes)	31.6% (6/19 test trials)	52.6% (10/19 test trials)	47.4% (9/19 test trials)	31.6% (6/19 test trials)
Javelin	Discus and Shot put	14.3% (1/7 athletes)	15.8% (3/19 test trials)	21.6% (4/19 test trials)	31.6% (6/19 test trials)	31.6% (6/19 test trials)
Discus	Shot put	14.3% (1/7 athletes)	36.8% (7/19 test trials)	52.6% (10/19 test trials)	52.6% (10/19 test trials)	42.1% (8/19 test trials)
Discus	Javelin	14.3% (1/7 athletes)	26.3% (5/19 test trials)	36.8% (7/19 test trials)	21.1% (4/19 test trials)	26.8% (5/19 test trials)
Shot put	Discus	14.3% (1/7 athletes)	47.4% (9/19 test trials)	57.9% (11/19 test trials)	47.4% (9/19 test trials)	31.6% (6/19 test trials)
Shot put	Javelin	14.3% (1/7 athletes)	42.1% (8/19 test trials)	47.4% (9/19 test trials)	47.4% (9/19 test trials)	36.8% (7/19 test trials)
Javelin	Discus	14.3% (1/7 athletes)	52.6% (10/19 test trials)	36.8% (7/19 test trials)	21.1% (4/19 test trials)	36.8% (7/19 test trials)
Javelin	Shot put	14.3% (1/7 athletes)	10.5% (2/19 test trials)	15.8% (3/19 test trials)	26.3% (5/19 test trials)	26.3% (5/19 test trials)

Similar results were found when the performance of SVM models was determined using the kinematic patterns of shot puts as test data. While the prediction accuracy is slightly lower when all variables are considered, the lowest prediction accuracy of 31.6% is larger than for the discus split and is achieved when all variables and only the lower-body variables are used as test data.

The lowest prediction accuracies are generally found when the SVM models are tested based on javelin throwing patterns (15.8–31.6%). When the SVM models for athlete-classification are trained on discus and shot put data, it seems to be more difficult to assign the movement patterns of javelin throwing to the individuals. Similar results can be observed in the pairwise cross-validations, which are also shown in Table 2.

In Table 3, the results of the discipline-classification are listed with the same variable partitions as in Table 2. When all variables were included in the discipline-classification, the respective disciplines could be predicted with an accuracy of 100%, based on the kinematic throwing patterns.

**TABLE 3:** Prediction accuracy of the discipline-classification with leave-athlete-out cross-validation for different data partitions.

Test data	Training Data	Random baseline	All variables	All variables (without throwing arm)	Only upper-body variables (without throwing arm)	Only lower-body variables
A1	A2–A7	33.3% (1/3 disciplines)	100.0% (9/9 test trials)	100.0% (9/9 test trials)	100.0 (9/9 test trials)	100.0% (9/9 test trials)
A2	A1 and A2–A7	33.3% (1/3 disciplines)	100.0% (8/8 test trials)	100.0% (8/8 test trials)	100.0% (8/8 test trials)	100.0% (8/8 test trials)
A3	A1–A2 and A4–A7	33.3% (1/3 disciplines)	100.0% (7/7 test trials)	100.0% (7/7 test trials)	85.7% (6/7 test trials)	100.0% (7/7 test trials)
A4	A1–A3 and A5–A7	33.3% (1/3 disciplines)	100.0% (7/7 test trials)	100.0% (7/7 test trials)	100.0% (7/7 test trials)	100.0% (7/7 test trials)
A5	A1–A4 and A6–A7	33.3% (1/3 disciplines)	100.0% (8/8 test trials)	100.0% (8/8 test trials)	100.0% (8/8 test trials)	100.0% (8/8 test trials)
A6	A1–A5 and A7	33.3% (1/3 disciplines)	100.0% (9/9 test trials)	100.0% (9/9 test trials)	77.8% (7/9 test trials)	100.0% (9/9 test trials)
A7	A1–A6	33.3% (1/3 disciplines)	100.0% (9/9 test trials)	88.9% (8/9 test trials)	88.9% (8/9 test trials)	88.9% (8/9 test trials)

## Discussion

The results of this study reveal that the kinematic patterns of the three throwing disciplines in the decathlon (shot put, discus throw, and javelin throw) could be distinguished independently of the athlete with a prediction accuracy of up to 100% (57 of 57 throws) using an automatic classification using machine learning (i.e., SVMs). In addition, prediction accuracies of up to 52.6% (10 of 19 throws) also indicate the persistence of individual throwing characteristics of athletes across different throwing disciplines. The results further suggest that individual throwing characteristics across disciplines are more pronounced in shot put and discus throwing than in javelin throwing. This finding demonstrates that the approach of classifying movement patterns using machine learning methods allows for the identification of athlete- and discipline-specific similarities in throwing patterns across different disciplines in high-performance athletes and suggests new ways to explore sports training in different disciplines.

In the following sections, we discuss the results in more detail. In the athlete-classification, the highest prediction accuracies by SVM models based on all variables except the throwing arm variables mean that every second shot put kinematic pattern was correctly assigned to the corresponding individual athlete when the SVM model was trained on the kinematic throwing patterns of all athletes in javelin and discus throw. Prediction accuracies over ∼50%, which are well above the random baseline of 14.3%, provide a strong indication that individual movement signatures can be detected in different movements (e.g., different throwing disciplines). The present findings reinforce previous studies that showed the uniqueness and persistence of individual movement patterns within various movements and support the call for a stronger focus on individual athletes or patients in sports and movement science (e.g., Horst et al., 2017b).

Note that the highest prediction accuracy is achieved using SVM models that consider all joint angle waveforms except the angles of the throwing arm. Comparatively lower prediction accuracies using SVM models that take into account all joint angle waveforms (including the ones of the throwing arm) might be traced to a slightly reduced individuality and a predominant expression of the discipline specificity in the throwing-arm, joint-angle waveforms. However, further research is needed to determine whether this lower prediction accuracy is due to the specificity of the disciplines or due to the variability in throwing arm movements. In this regard, a joint angle-wise classification and determination of movement variability could provide further clarification.

Higher prediction accuracies for SVM models based on the joint angles waveforms of the upper body without the throwing arm in shot put and discus throwing provide evidence for increased individuality of the movement of the left arm, trunk, and head in comparison to the waveforms of the lower-body joint angles, which are more restricted by their contact to the ground. Whether lower prediction accuracies of the SVM models based on lower-body joint angles are only due to the comparably coarse biomechanical data acquisition without anatomical markers or due to the small geometric differences in the leg movements cannot be resolved satisfactorily here.

Considerably lower prediction accuracies of SVM models for athlete-classification that were trained with the kinematic patterns of shot put and discus throw and tested with javelin throws are in line with findings of national (Kunz, 1980) and international (Pavlović and Idrizović, 2017) decathletes, who showed a high correlation between performances in shot put and discus throwing, but no linear correlation with performance in javelin throwing. The finding that the individual throwing characteristics across the disciplines are more pronounced in shot put and discus throwing than in javelin throwing gives rise to the speculation about a more individual coupling of the joint angles of the trunk and lower body with the left arm and head in shot put and discus throwing. Future research is necessary to investigate whether cross-disciplinary individual characteristics in shot put and discus throwing also foster a positive transfer from training in one discipline to the other. An analysis of individual muscle activation signatures (Hug et al., 2019) during shot putting, discus, and javelin throwing could provide interesting insights in this context.

In discipline classification, a prediction accuracy of 100% for most cross-validation splits and combinations of considered variables implies an automatic and differentiated recognition of shot put, discus, and javelin throwing movement patterns. The results provide promising evidence for the ability of pattern recognition approaches using machine learning methods to distinguish between different qualities of whole-body movements (Schöllhorn and Bauer, 1997; Schorer et al., 2007).

Finally, some specificities of this pilot study should be kept in mind. The chosen pattern recognition approach based on probabilities relative to the number of choices is distinguished from null-hypothesis-testing approaches. No claims for generalization are made. In addition, the demand for a relatively high level of performance in different sports disciplines limited the possibilities for empirical data collection enormously. Some limitations arise from selecting decathletes on their way from juvenile to adult competition classes as the object for this pilot study. The athletes’ age suggests that some may not have completed puberty, and ongoing physical growth could have an additional influence on the consistency of their movement patterns. To what extent incomplete physical growth influences throwing patterns and throwing consistency requires further research.

## Conclusion

The results offer evidence for the possibility of automatic recognition of kinematic movement patterns originating from different sports disciplines and confirm the assumption of a strong and cross-disciplinary importance of individuality in at least two of the throwing disciplines investigated. That certain individual movement characteristics can be identified in the kinematic patterns of both shot put and discus throwing is intriguing. This finding must be distinguished from the recognition of an individual athlete within a single discipline, as shown for discus (Bauer and Schöllhorn, 1997) and javelin throwing (Schöllhorn and Bauer, 1998). An extension of this approach to the kinematic movement patterns in other sports disciplines such as the tennis serve, handball throw, or volleyball smash is reasonable. Exploring the respective proportion of individual characteristics in movement patterns in more detail, even for dissimilar movement classes, will be a challenge for future research. This exploration can be compared with the search for analogies between different biometric characteristics.

A further criterion for individuality, which could be summarized by homomorphism, could be added to the necessary criteria of uniqueness and persistence. Different from static biometric measures such as fingerprints, facial characteristics, or ear shapes, which are frequently directly related to static genetics, movement-based biometry is subject to dynamic changes and uncertain associations to the genome. While it is difficult to find a common underlying basis for the biometrics of finger, face, or ear apart from genetics, the comprehension of individual commonalities in different movements (e.g., throwing disciplines) could provide access to the underlying individuality of central nervous physiology and structure. Future applications of this approach could investigate the extent to which the central nervous system or the muscle physiology are modifiable beyond an individual’s range.

Against this backdrop, the probability of finding a single (time-independent) optimal movement pattern for an individual athlete is more than challenging. Instead, rethinking the understanding of an optimal movement pattern is promising. An extension of the term “optimal” by situation-optimal, as the currently optimal solution for an individual athlete, may be initially tempting. However, an optimal solution would only serve as a theoretical model and could never be realistically achieved. Because the motor system of an individual is constantly changing and adapting, the model of a situation-optimal movement pattern would also have to constantly change and adapt. Alternatively, the assumption of a situation-optimal model that is constantly changing could be more advantageous for motor learning than for the pursuit of an insurmountable goal.

The study showed that an applied pattern recognition approach based on a machine learning classification provides an alternative and holistic approach for the analysis of biomechanical movement data. This approach is closely connected to a statistical method based on the original concept of probabilities and may help to circumvent some of the limitations connected with the Fisher and Mackenzie (1923) and Neyman and Pearson (1928) statistics.

Taken together, the findings of human movement science regarding the uniqueness and persistence of individual movement patterns based on machine learning methods and the insights into the influencing factors indicated in this study suggested that we are still at the beginning of understanding the individuality of moving and learning human beings.

## Data Availability Statement

The raw data supporting the conclusions of this article will be made available by the authors, without undue reservation, to any qualified researcher.

## Ethics Statement

Ethical review and approval was not required for the study on human participants in accordance with the local legislation and institutional requirements. Written informed consent for participation was not required for this study in accordance with the national legislation and the institutional requirements.

## Author Contributions

All authors contributed to the article, critically revised the manuscript, and approved the final version. WIS designed the experiment. DJ, HB, and WIS conducted the acquisition and processing of the data. FH, DJ, and HB analyzed the data. FH, DJ, and WIS designed the figure. FH and WIS interpreted the data and wrote the manuscript.

## Conflict of Interest

The authors declare that the research was conducted in the absence of any commercial or financial relationships that could be construed as a potential conflict of interest.
